# The Relationship Between Posterior Tibial Slope and Knee Range of Movements in Posterior Stabilized Total Knee Replacement: A Navigation-Assisted Analysis of 120 Cases

**DOI:** 10.7759/cureus.29695

**Published:** 2022-09-28

**Authors:** Niranj G Radhamony, Anant Chaugule, Harish S Bhende

**Affiliations:** 1 Trauma and Orthopaedics, Royal Stoke University Hospital, University Hospitals of North Midlands NHS Trust, Stoke-on-Trent, GBR; 2 Orthopaedics, Ortho One Orthopaedic Clinic, Sangli, IND; 3 Orthopaedics, Laud Clinic, Mumbai, IND

**Keywords:** navigation-assisted tkr, mediolateral laxity, residual flexion, posterior stabilized tkr, range of movement, posterior tibial slope

## Abstract

Background

Posterior tibial slope (PTS) is one of the factors that determine the postoperative range of movement (ROM) in total knee replacement (TKR). While biomechanical factors influencing ROM such as PTS, soft-tissue balancing, and choice of implants are surgeon-dependent, non-biomechanical factors such as physiotherapy and pain are subjective and beyond the surgeon’s control. Using navigation, we avoided these factors and objectively correlated the difference in PTS with ROM.

Methodology

A total of 120 cases of posteriorly stabilized (PS) TKR were included. The X-ray-measured difference in PTS was compared to the corresponding change in the ROM intraoperatively using the TKR navigation system. Based on the change in PTS, the cases were classified into three groups, and the intergroup variability of mean postoperative ROM, mediolateral laxity difference (MLD), and residual flexion (RF) was calculated.

Results

An average MLD of 1.39 mm in extension and 1.79 mm in flexion and an average RF of 3.18 degrees were seen. This uniformity neutralizes the effects of these factors on the ROM. The mean difference in the PTS in the three groups was 4.45, 10.76, and 17.98 degrees, and the mean change in the ROM was 3.07, 1.47, and 2.84 degrees, respectively. There was no statistically significant change in ROM with that of change in PTS.

Conclusions

In our study, it was shown statistically that the coronal and sagittal planes were uniformly balanced using navigation assistance, and the PTS and the postoperative ROM were the only variables. The correlated results showed that a change in the PTS does not affect the postoperative ROM in PS TKR using the implant system used in our study.

## Introduction

Total knee replacement (TKR) is a well-accepted procedure for the management of advanced arthritis of the knee, providing pain relief and adequate knee movements to perform daily activities [1]. Hence, achieving the maximum knee range of movement (ROM) after TKR is desirable for patients and is expected of surgeons. The biomechanical factors that affect ROM after TKR include posterior tibial slope (PTS), implant design, posterior femoral condyle offset (PFCO), soft-tissue balancing, and the size of the implant replacing the joint space [2-4]. While these factors can be modified by the surgeon, there are several postoperative factors that influence the ROM such as physiotherapy [5], arthrofibrosis [6], patient motivation, pain control, and infection that are unpredictable and not fully under the control of the surgeon.

Among the various biomechanical factors, the contribution of PTS in determining ROM remains a subject of debate. Some studies have shown a positive correlation between the two variables [4,7], whereas others have shown none [3]. We aimed to study the influence of PTS on knee ROM in TKR when other influencing factors were neutral. Moreover, recording knee ROM immediately before and after TKR using navigation would be equivalent to a cadaveric study as it would represent an objective measurement of these values using navigation. In addition, we aimed to analyze whether the other intraoperative biomechanical factors such as residual flexion (RF) and soft-tissue balancing are statistically uniform by the use of navigation and statistical analysis. Hence, the only variable would be the difference in ROM and the difference in PTS.

Based on the results of a few earlier studies on ROM in TKR [4,7], we hypothesized that a significant reduction in the PTS will cause a corresponding significant reduction in ROM after surgery. Therefore, we recorded the preoperative and the postoperative PTS to calculate the difference in the PTS obtained during the surgery. In this analytical study, we calculated the change in ROM following TKR and compared it to the change in PTS during the procedure.

## Materials and methods

A total of 120 TKRs in 96 patients performed at our center using navigation assistance were included in the analysis. The study included cases from March 2018 to March 2019. Posteriorly stabilized (PS) rotating platform implants (PS 150™ and Attune™, Depuy™, Johnson & Johnson™) and computer navigation using the Brainlabs™ system were used for all cases. Knees with gross bony irregularities, defects, or extra-articular deformities where precise radiological measurements were not possible were excluded. An institutional and ethical review board approval was not considered as the study is a retrospective observational analysis of the data routinely recorded while performing the navigation-assisted TKR.

**Figure 1:**
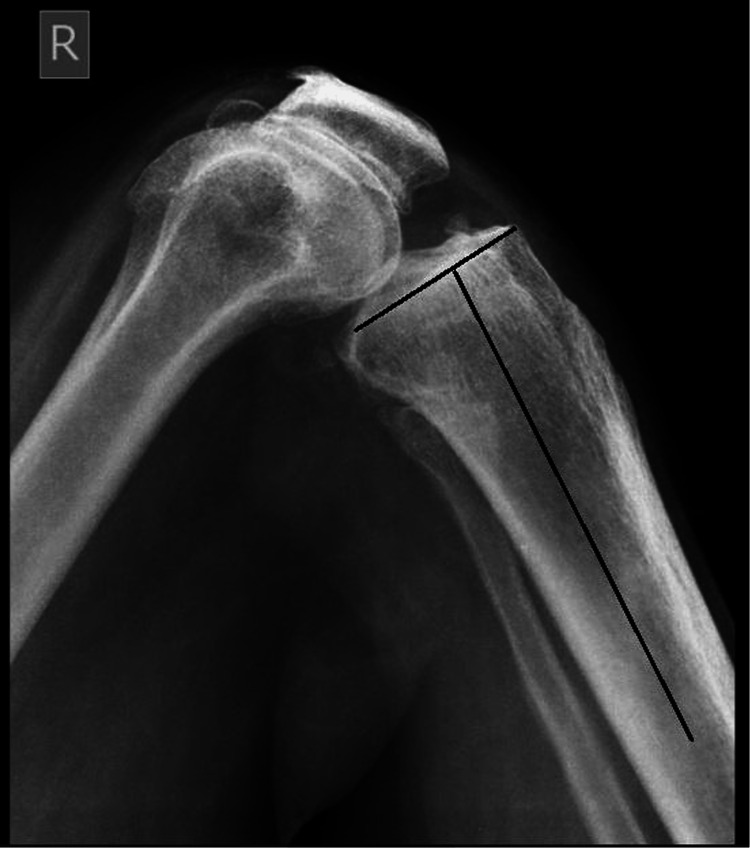
Measurement of preoperative tibial slope from the lateral view of the X-ray.

The preoperative and postoperative PTS were measured from the digital radiographs by two orthopedic surgeons, and the average of the two values was taken for the analysis. Lateral views of the knees were taken in such a manner that both the femoral condyles superimposed each other and had 15 cm of proximal tibia included in the film. Two lines were drawn as follows: line 1 was drawn by connecting the midpoints of the outer cortical diameter at 5 cm and 15 cm distal to the knee joint, and line 2 was drawn tangential to the tibial plateau in the preoperative radiographs and parallel to the tibial base plate in the postoperative radiographs [8]. The acute angle between the two lines was measured and subtracted from 90 degrees which gave the tibial slope (Figure 1). These measurements were performed digitally using open-source software, Image J™.

All cases were operated on by the same surgeon (supervising author). After the placement of navigation arrays over the distal femur and proximal tibia, the hip center point, the distal femur mechanical axis point, the proximal tibial mechanical axis point, and the lateral and medial malleolar points were registered. The free maximum flexion was noted using navigation before the surgery by lifting the thigh vertically and allowing the knee to flex freely under gravity. TKR was then performed using the navigation technique as per the Brainlabs™system. As the implants used were of rotating platform type, it was aimed at creating a slope close to 0 degrees [8] with the help of the navigation surface probe.

At the end of implantation, the medial and lateral laxities were numerically assessed by monitoring the medial and lateral gaps from the navigation monitor after giving valgus and varus stress, respectively, to the knee in full extension and in 90-degree flexion. In all cases, balancing was done in the recommended manner using navigation for the implant in such a way that the difference between medial and lateral laxities (MLD) after implantation was within 1-3 mm. This was later statistically analyzed to check for uniformity among cases to make difference in PTS and difference in ROM as the variables. Similarly, residual flexion (RF) at the end of implantation was aimed at about 3-5 degrees for all cases as per the recommendations of the navigation system. This could indicate that at the end of implantation, the tissue tension was maintained uniformly, which may otherwise affect the knee ROM. After final implantation, the postoperative maximum free flexion was routinely recorded in the same manner which was used to record the preoperative ROM.

Statistical analysis

The observed values were tabulated, coded, and analyzed using SPSS, version 17 for Microsoft Windows (IBM Corp., Armonk, NY, USA). Pearson correlation between the reduction in the PTS and the difference between the preoperative and postoperative knee ROM was calculated.

The cases were divided into three groups (group I: 0-7.5 degrees, group II: 7.6-15 degrees, and group III: 15.1-22.5 degrees) based on the difference between preoperative and postoperative PTS. Postoperative flexion, postoperative RF, and the mediolateral laxity difference (MLD) at the end of implantation were compared among the three groups using analysis of variance. P-values of <0.05 were considered statistically significant for all analyses.

## Results

A total of 120 patients were included in the analysis. The respective preoperative and postoperative mean PTS, ROM, and the difference between the preoperative and postoperative values are listed in Table 1.

**Table 1:** Summary of average PTS and ROM values. PTS: posterior tibial slope; ROM: range of movement; N: total number of cases

	N = 120
Average preoperative PTS	12.76°
Average postoperative PTS	2.68°
Average preoperative ROM	119.80°
Average postoperative ROM	117.79°
Average difference in PTS	10.10°
Average difference in ROM	2.01°

**Figure 2:**
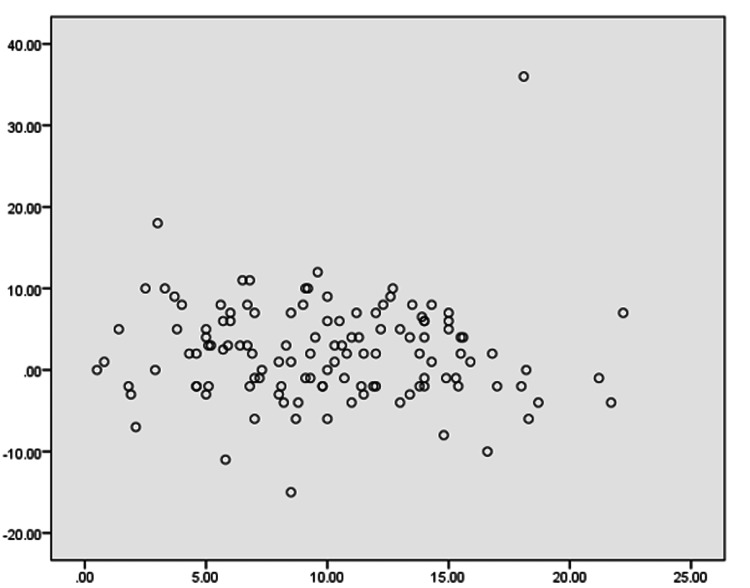
Scatter plot showing the correlation between the difference in PTS and the difference in ROM (x-axis showing the difference in PTS in degrees; y-axis showing the difference in ROM in degrees). PTS: posterior tibial slope; ROM: range of movement

A scatter plot based on our analysis (Figure 2) showed the correlation between the difference in PTS and the difference in ROM (x-axis showing the difference in PTS; y-axis showing the difference in ROM). The plot showed that the change in ROM did not have a correlation with the change in PTS.

The MLD was 1.39 mm for extension and 1.79 mm for 90° flexion. There was no statistically significant deviation of this value from the mean for all cases. Similarly, the RF at the end of implantation was aimed at 0-5° for all cases. The mean RF obtained was 3.18°, and the cases had no significant deviation from the mean value.

The change in the PTS from the preoperative to the postoperative values did not show any statistically significant corresponding change in the ROM measurements (Table 2).

**Table 2:** Correlation between the difference in flexion and difference in PTS. ROM: range of movement; PTS: posterior tibial slope; N: total number of cases

		Difference in ROM	Difference in PTS
Difference in ROM	Pearson correlation	1	-0.110
	Significance (two-tailed)		0.233
	N	120	120
Difference in PTS	Pearson correlation	-0.110	1
	Significance (two-tailed)	0.233	
	N	120	120

Using Pearson correlation, the p-value for the analysis was -0.110. There was no statistically significant variability of the analyzed parameters (MLD in flexion, MLD in extension, and RF) among the three groups, implying that these factors were uniform among cases, which might otherwise skew the ROM values. The corresponding analysis is presented in Table 3.

**Table 3:** Intergroup comparison of ROM, RF, and MLD. PTS: posterior tibial slope; ROM: range of movement; RF: residual flexion; MLD: mediolateral laxity difference; SD: standard deviation

Groups (difference in PTS)	Number (total 120)	Flexion difference (difference in ROM)			RF			MLD in extension			MLD in flexion		
		Mean	SD	P-value	Mean	SD	P-value	Mean	SD	P-value	Mean	SD	P-value
Group I: 0–7.5	32	3.07	5.97	0.381	2.79	2.76	0.528	1.15	0.96	0.745	1.73	1.95	0.730
Group II: 7.6–15	70	1.47	3.38		3..38	2.24		1.55	3.08		1.79	1.69	
Group III: 15.1–22.5	16	2.84	9.73		3.00	3.14		1.31	0.96		2.15	2.07	

## Discussion

Postoperative ROM is an important factor that determines the success of TKR. Among the variables that determine postoperative ROM, PTS has been considered to be one of the most biomechanically important factors [9]. Some studies correlate PTS positively with postoperative ROM, whereas others imply no correlation. According to one study, the influence of the change in PTS on ROM was greater with cruciate retaining (CR) TKR than with PS TKR [10]. Bellemans et al. assessed cadaver simulations of PCL-retaining TKR in 21 cases with the use of a three-dimensional computer model. He showed that a 1-degree increase in the PTS would correspondingly increase flexion by 1.7 degrees [11]. Some studies have also suggested that an increase in PTS could increase the maximal knee flexion after PS TKR [2,7,12]. However, a study by Kansara et al. reported no correlation between a change in PTS and knee ROM in PS TKR [3]. The contrasting results of these studies could be because postoperative ROM is not only dependent on biomechanical factors but also on other non-biomechanical factors. Whereas biomechanical factors such as PTS, PFCO, and soft-tissue balancing are modifiable by the surgeon, the non-biomechanical factors such as physiotherapy, patient’s motivation, pain, and arthrofibrosis that can affect the ROM measurements are not under the control of the surgeon or the investigator. According to Shoji et al. [13], postoperative physiotherapy has a significant influence on ROM after TKR. Lam et al. [6] showed that postoperative arthrofibrosis and contracture can alter the ROM significantly following TKR. The strength of our study is that other than PTS, other external factors were found to be statistically uniform, and the direct influence of a change in the PTS on the ROM was assessed using navigation similar to an in-vitro study.

Another important factor to be considered in our analysis is the mode of ROM measurements. Some of the commonly used modalities include the use of clinical goniometers, radiographic measurements, and, more recently, the use of mobile phone applications [14,15]. Measurements using a clinical goniometer are shown to be inaccurate and less reproducible [16,17], and the usage of radiographs for the measurement of ROM requires routine true lateral full flexion and full extension radiographs of the knee [8]. According to one study, the disparity in the values of ROM measurements between observers could be due to measurement parallax, level of education, or the years of experience of the observer. The study also suggested that the limbs of the goniometer have to be long enough to improve the accuracy of measurements [14]. Edwards et al. reported that among various observers, the error in knee ROM measurement could be between +15 degrees and -15 degrees [18]. The recent trend toward the usage of mobile applications for the same has shown more promise by achieving better reproducibility [17]. However, all these methods are biased by the amount of force given passively or actively to attain maximum flexion [14,19]. In an analysis, when we aim to relate the PTS variability with that of the ROM, an accurate, objective, and reproducible method of recording ROM is desirable. Therefore, we obtained ROM measurements before the procedure and after implantation using navigation which is accurate and reproducible to 0.1 degrees, as per the Brainlabs™ computer system. Under anesthesia, the leg was allowed to flex freely under gravity as the thigh was lifted vertically eliminating the bias due to any external force to flex the knee. Postoperative external factors as already mentioned are also eliminated. To our knowledge, there are no previous studies that have used such a method of recording ROM.

For measuring PTS, we used preoperative and postoperative lateral radiographs. Various methods of measurement have been suggested by various authors in a true lateral radiograph of the knee. We followed the already mentioned method, as suggested by Yoo et al. [8]. Even though we also recorded the postoperative slope using a navigation surface probe after making the desired tibial cuts intraoperatively, we used the postoperative radiographs for the analysis to maintain the uniformity of the measurement method.

The mean preoperative PTS in our study was 12.76 degrees. Mohanty et al. [20] reported a mean PTS of 11.64 degrees and Nekkanti et al. [21] reported an average of 10.37 degrees in Indian cases. The mean postoperative PTS in our study was 2.83 degrees.

In our study, the average preoperative and postoperative flexion angles measured using computer navigation were 119.80 and 117.79 degrees, respectively. On analyzing the difference in PTS with the difference in flexion angle, no statistical significance was noted (p = -0.33). Our results correlated with Kansara et al. who also reported no significant relationship between the PTS and ROM in PS TKR [3]. However, in his study, the ROM measurements were made clinically and not using navigation. Hence, it is evident from the above findings that the variables indirectly quantifying soft-tissue tension that could affect ROM [22,23] were maintained uniformly, and their influence on ROM was neutralized.

Our study has certain limitations. First, we performed the retrospective analysis using only a single implant manufacturer (Depuy™). Therefore, the results of this study may not represent TKR performed using other systems. Second, regarding the method of performing TKR, all cases were performed using the PS method; hence, the results of the study cannot necessarily apply to the CR method.

## Conclusions

The findings of our study conclude that PTS has no influence on the ROM in PS TKR using the included implant. Our study also proposes navigation assistance as a method to record the ROM difference by negating the non-biomechanical factors that affect the postoperative ROM.
